# Distal Cervical Carotid Artery Dissection after Carotid Endarterectomy: A Complication of Indwelling Shunt

**DOI:** 10.1155/2010/816937

**Published:** 2010-08-04

**Authors:** Tomonori Tamaki, Node Yoji, Norihiro Saito

**Affiliations:** Department of Neurosurgery, Nagayama Hospital, Nippon Medical School, 1-7-1 Nagayama Tama-shi, Tokyo-to 206-8512, Japan

## Abstract

The technical factors and surgical methods employed in carotid endarterectomy are controversial. In particular, whether or not to use an indwelling arterial shunt during carotid endarterectomy remains a source of conflict. We describe a rare case in which uncomplicated carotid endarterectomy was followed by distal internal carotid artery dissection and suggest that this devastating complication was due to intimal damage produced by the use of an indwelling arterial shunt.

## 1. Introduction

The merits and demerits of using an arterial shunt during carotid endarterectomy (CEA) have long been a topic that provokes discussion and controversy among cerebrovascular surgeons [1–4]. Potential complications of shunt placement include particulate embolization of atheromatous material, either distal to the shunt tip or through the shunt itself, as well as intimal damage that may lead to carotid dissection [1–4]. Although the latter possibility has been raised by numerous authors, we are aware of only one case in which distal carotid artery dissection was documented as being due to a shunt [5]. We present another rare case of internal carotid artery dissection caused by an indwelling arterial shunt. 

## 2. Case Report

**Figure 1:**
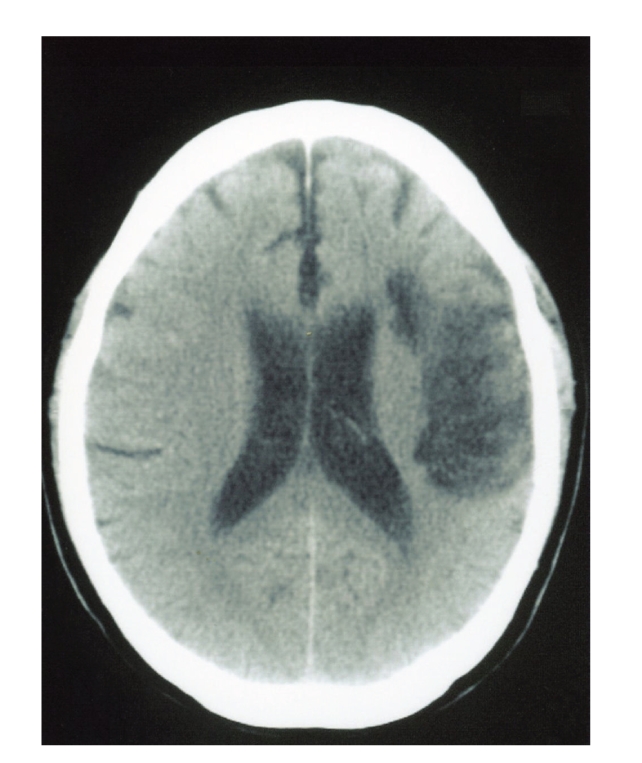
Axial Computed tomography of the brain demonstrated cortical infarction in the left frontal lobe.

**Figure 2:**
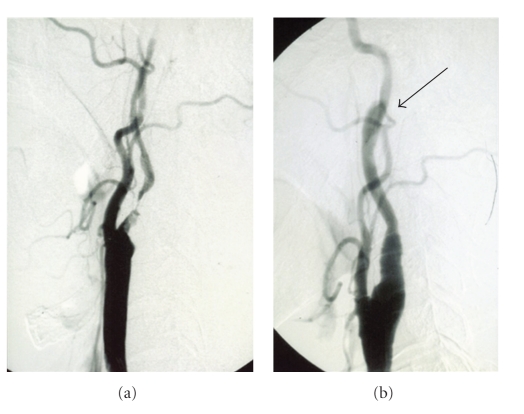
(a) Preoperative left carotid angiogram showing severe stenosis of the left internal carotid artery. (b) Postoperative left carotid angiogram showing dissecting formation (arrow) at the several centimeters distal to the operative site and improvement of stenosis.

**Figure 3:**
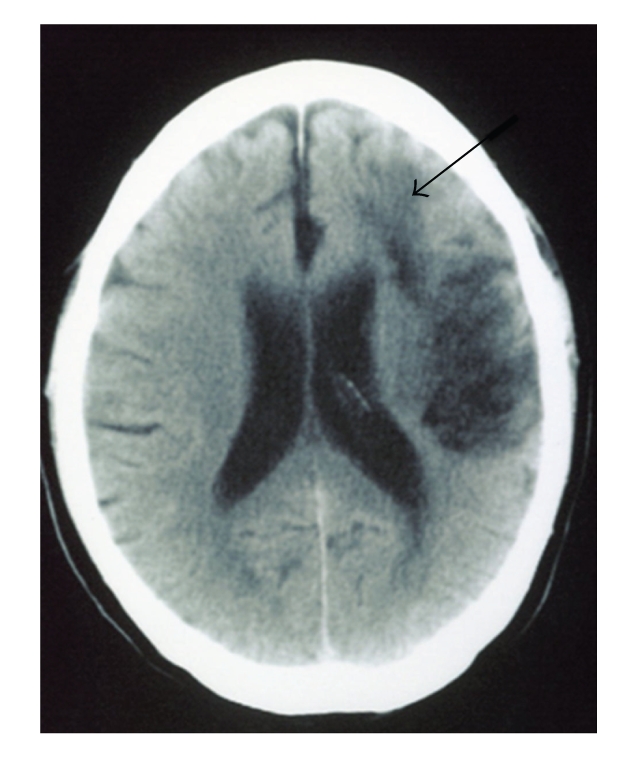
Axial Computed tomography of the brain 13 days after the carotid endarterectomy, demonstrated a new infarct in the white matter of the left frontal lobe (arrow).

A 72-year-old right-handed man was admitted to our institution. Four days before admission, he had experienced transient right hemiparesis for 20 minutes. On examination, he was alert and fully oriented. Muscle power and sensation were normal. Computed tomography (CT) of the brain demonstrated cortical infarction in the left frontal lobe, Figure 1. He was started on medical management with antiplatelet therapy. After 13 days, angiography was performed and revealed severe stenosis of the left internal carotid artery (ICA) from the bulb and extending 5 cm distally. Other lesions were not detected (Figure 2(a)). At 35 days after the stroke, CEA was performed under general anesthesia as monitoring of somatosensory evoked potential (SSEPs). Patient's hesitation for operation delayed CEA. Routine CEA was performed, and the arteriotomy was repaired with a 7-0 prolene suture and patch angioplasty. During arterectomy, we used an indwelling shunt routinely (Pruitt-Inahara Carotid Shunt, LeMaitre Vascular). The shunt was easily advanced from the common carotid artery, to the internal carotid artery and no changes of SSEPs were noted at any time. The patient recovered from surgery without any neurological deficits. CT scanning of the brain 1 and 3 days after the operation showed no new lesions. 13 days after the operation, he developed the acute onset of expressive aphasia with inability to follow commands and worsening paresis of the right arm. Emergency CT revealed a new infarct in the white matter of the left frontal lobe, Figure 3. Angiography showed arterial dissection at the distal side of the carotid endarterectomy site (Figure 2(b)).

## 3. Discussion

Use of an arterial shunt during CEA is a controversial subject. Arguments against universal shunting focus on the added morbidity that shunting may, citing evidence create the following. (1) Stroke rates are higher in shunted than nonshunted patients (especially in shunted patients without any evidence of intraoperative ischemia), (2) shunts may fail intraoperatively, (3) shunting can directly cause arterial injury, and (4) good results are reported by surgeons who never use shunts [1–4]. However, there are very few well-documented case reports of shunt failure. Cervical carotid artery dissection is often spontaneous or idiopathic, but it is also associated with trauma to the carotid arteries and with catheterization (as in percutaneous carotid angiography) [5–8]. The mechanism of injury in these latter cases is presumed to be an intimal tear that allows a new dissection plane to be established in the arterial wall [5–7]. Intraoperative use of an indwelling shunt may cause direct injury to the arterial wall. In our case, catheter manipulation for angiography and shunt tube placement were suspected to be the cause(s) of arterial wall injury. Considering the timing of onset of the patient's devastating neurological sequelae, the most likely cause of arterial dissection was shunt placement during CEA. We are aware of only one previous case in which distal carotid artery dissection was documented as being due to shunting [5]. Loftus reported a case of carotid artery dissection after CEA, but his case differed from ours in two respects: (1) repeated carotid arterectomy was required for evacuation of thrombus after CEA and, (2) the shunt used was not designed exclusively for CEA [5]. Our patient is the first reported case with carotid artery dissection after CEA caused by an indwelling arterial shunt.
